# A new method for isolating and analysing coccospheres within sediment

**DOI:** 10.1038/s41598-020-77473-5

**Published:** 2020-11-26

**Authors:** Beth Langley, Paul R. Halloran, Ann Power, Rosalind E. M. Rickaby, Prabhjoat Chana, Poppy Diver, David Thornalley, Christian Hacker, John Love

**Affiliations:** 1 Geography, College of Life and Environmental Sciences, University of Exeter, Exeter, EX4 4RJ UK; 2 Biosciences, College of Life and Environmental Sciences, University of Exeter, Exeter, EX4 4QD UK; 3 Department of Earth Sciences, University of Oxford, South Parks Road, Oxford, OX1 3AN UK; 4 Luminex B.V., Het Zuiderkruis 1, 5215 MV ‘s-Hertogenbosch, The Netherlands; 5 Department of Geography, University College London, London, WC1H 9LG UK

**Keywords:** Ocean sciences, Marine biology, Marine chemistry, Palaeoceanography, Biogeochemistry, Biooceanography, Palaeoecology, Carbon cycle

## Abstract

Size is a fundamental cellular trait that is important in determining phytoplankton physiological and ecological processes. Fossil coccospheres, the external calcite structure produced by the excretion of interlocking plates by the phytoplankton coccolithophores, can provide a rare window into cell size in the past. Coccospheres are delicate however and are therefore poorly preserved in sediment. We demonstrate a novel technique combining imaging flow cytometry and cross-polarised light (ISX^+PL^) to rapidly and reliably visually isolate and quantify the morphological characteristics of coccospheres from marine sediment by exploiting their unique optical and morphological properties. Imaging flow cytometry combines the morphological information provided by microscopy with high sample numbers associated with flow cytometry. High throughput imaging overcomes the constraints of labour-intensive manual microscopy and allows statistically robust analysis of morphological features and coccosphere concentration despite low coccosphere concentrations in sediments. Applying this technique to the fine-fraction of sediments, hundreds of coccospheres can be visually isolated quickly with minimal sample preparation. This approach has the potential to enable rapid processing of down-core sediment records and/or high spatial coverage from surface sediments and may prove valuable in investigating the interplay between climate change and coccolithophore physiological/ecological response.

## Introduction

Coccolithophores are a highly abundant and ubiquitous group of single-celled phytoplankton that are a critical component of global biogeochemical cycles^1^. The importance of coccolithophores to global biogeochemical cycles comes in part from their production of a ‘coccosphere’, a calcium carbonate exoskeleton^2^. Coccospheres are comprised of highly complex delicate plates called coccoliths^3^. During the diploid, non-motile and autotrophic phase, coccolithophores produce heterococcoliths that interlock their distal and proximal shields to form a coccosphere that surrounds the cell^4^. Most phytoplankton are not preserved in the fossil record because they have no hard parts, however, the calcified (calcium carbonate, CaCO_3_) exoskeletons of coccolithophores sink to the ocean floor where they become preserved in the sedimentary record^5^. Coccolithophore remains therefore provide a window into past ecosystem function^6^.

Fossil coccospheres provide a wealth of information on cellular traits that can be related directly to the former living coccolithophore, including cell size and shape, number of coccoliths per cell and arrangement of coccoliths^7^. Cell size is a fundamental master trait that influences virtually every aspect of phytoplankton biology at the cellular, population and community levels^8^. Physiological and ecological processes that scale with size include metabolic rates, nutrient uptake and diffusion, cellular composition, sinking velocity, grazing, population abundance, trophic interactions and diversity^9^. These fossils are therefore a valuable archive of information that can inform many aspects of marine ecology and biogeochemistry, including coccolithophore growth rates^10^ and response to changes in palaeoenvironmental conditions^6,11^.

Preservation of coccospheres in sediments is generally uncommon^7^. Unlike other plankton groups that have hard parts, such as foraminifera, diatoms, and radiolarians, for which the entire ‘skeleton’ is a single unit and therefore more readily preserved^12–14^, coccospheres often disintegrate into individual coccoliths once the cell and organic binding material are lost^7^. Fossil coccolithophore studies are therefore largely based on disaggregated coccoliths as this is the most common state in which coccolithophore remains are found^15,16^. Important information can be obtained from individual coccoliths, such as trends in diversity, evolution, biogeography, and palaeoceanographic conditions^17–20^, however, to derive reliable cellular level information we must have an understanding of the coccospheres from which they are derived^11^. The scarce abundance of intact coccospheres limits our current cellular level understanding to geographical regions where coccosphere preservation is common^6,10,11,20–25^.

A major challenge in reconstructing past cell size from coccospheres is the detection of coccospheres from large sediment samples where they are rare ‘events’, diluted within the sedimentary matrix by minerogenic particles and other calcareous fragments. Traditional techniques to analyse fossil coccospheres rely on time-consuming light microscopy of smear slides^11^. In more recent years, techniques such as flow cytometry have been employed to isolate coccoliths from clay^26^ and automatic recognition of coccoliths by neural networks have been developed to automate the identification of coccoliths^27^. Here, we introduce imaging flow cytometry as a novel technique for rapid, high-throughput analyses to identify, visually isolate and morphologically characterise fossil coccospheres from marine sediments, that delivers detailed quantitative data about coccolithophore morphology.

Imaging flow cytometry combines flow cytometry and light microscopy, enabling rapid morphological and fluorescence analysis of high sample numbers. Here, we use the ImageStream Mk II (ISX; Luminex Corp. Seattle, US). The ISX hydrodynamically focuses objects (~ < 100 µm) sequentially into a narrow stream allowing for individual particle interrogation via lasers and focused light, whilst time delay integration cameras capture images of each object. Objects can be acquired at rates of up to 4000 objects per second at 20X, 40X and 60X magnifications. Data is processed and analysed using the IDEAS software (Luminex Corp) to generate high-resolution images and graphical and statistical data regarding fluorescence, scatter of light, morphological features, and concentration data.

The ISX is primarily used for biomedical research, only recently has it been employed for phytoplankton studies^28–30^. We present the first application of imaging flow cytometry to analyse phytoplankton fossils and introduce a novel method that combines imaging flow cytometry and cross-polarised light (ISX^+PL^) to detect coccospheres based on their distinctive birefringent properties. Distinguishing coccospheres from marine sediment using ISX^+PL^ requires that coccospheres have defined and contrasting optical and/or morphological properties to ‘background’ marine sediment. Coccoliths are made from calcite, a highly birefringent mineral (birefringence index = 2.16)^31^. Exploiting this extreme birefringence of coccospheres, relative to other components of marine sediments, such as clay^26^, provides a basis for effective visual isolation of coccospheres from non-birefringent background sediments. Distinct morphological properties of coccospheres can then be used to distinguish coccosphere populations from other birefringent material.

By utilising birefringence properties and classifying the morphological characteristics of a cultured coccolithophore sample and marine sediment with coccolithophore material removed, we constructed a protocol and template to allow the visual isolation of coccospheres from marine sediment. The protocol was applied to marine sediment from a North Atlantic core to distinguish coccospheres and extract morphological information that will enable the reconstruction past coccolithophore cell size.

## Results

### Reconstructing cell size via ISX^+PL^

**Figure 1:**
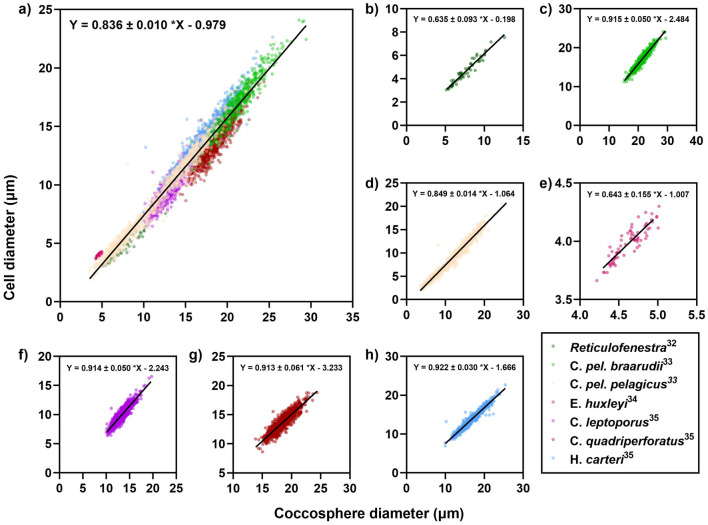
Coccosphere diameter and cell diameter relationship for typical placolith coccolithophores. (**a**) Coccosphere and cell diameter measurements for typical placolith coccolithophores with linear regression line of all species plotted in black (R^2^ = 0.956, p < 0.0001). Uncertainty of the slope represents the 95% confidence interval. (**b**–**h**) Coccosphere and cell diameter measurements with linear regression line plotted in black for (**b**) fossilised *Reticulofenestra*^32^ (R^2^ = 0.933, p < 0.0001) (**c**) cultured *C*. *pel. braarudii*^33^ (R^2^ = 0.913, p < 0.0001) (**d**) cultured, trap sample and fossilised *C*. *pel. pelagicus*^33^ (R^2^ = 0.975, p < 0.0001) (**e**) cultured *E*. *huxleyi*^34^ (R^2^ = 0.802, p < 0.0001) (**f**) cultured *C*. *leptoporus*^35^ (R^2^ = 0.834, p < 0.0001) (**g**) cultured *C*. *quadriperforatus*^35^ (R^2^ = 0.805, p < 0.0001) (**h**) cultured *H*. *carteri*^35^ (R^2^ = 0.939, p < 0.0001).

To investigate the relationship between coccosphere diameter and cell diameter to determine the applicability of ISX^+PL^ to reconstruct cell size, we examined published data^32–35^ representing individually measured coccosphere and cell diameters from a range of typical placolith-type species (Fig. 1). This sub-group is the most ecologically significant and produces coccospheres that are preserved in the fossil record^7^. Coccosphere diameter is a general proxy for cell diameter of placolith coccolithophores, demonstrated by a statistically significant correlation between these two parameters (R^2^ = 0.956, p < 0.0001). The slope of the linear regression line is 0.836 with an uncertainty of ± 0.01 µm at the 95% confidence interval. However, on a species-level, we found a significant difference between linear regression lines of individual species (F = 21.74, p < 0.0001) (Fig. 1b–h). This introduces potential error in cell size estimates which we explore in the discussion.

### ISX^+PL^ method development

Distinguishing coccospheres from marine sediment using imaging flow cytometry requires that coccospheres have distinct and contrasting optical and/or morphological properties to other marine sediment particulates. We exploited the birefringence of coccospheres, as demonstrated by Halloran et al.^26^ to omit a considerable proportion of marine sediment and utilised the morphological properties of coccospheres to distinguish them from other birefringent material.

### Coccosphere detection using birefringent properties

**Figure 2:**
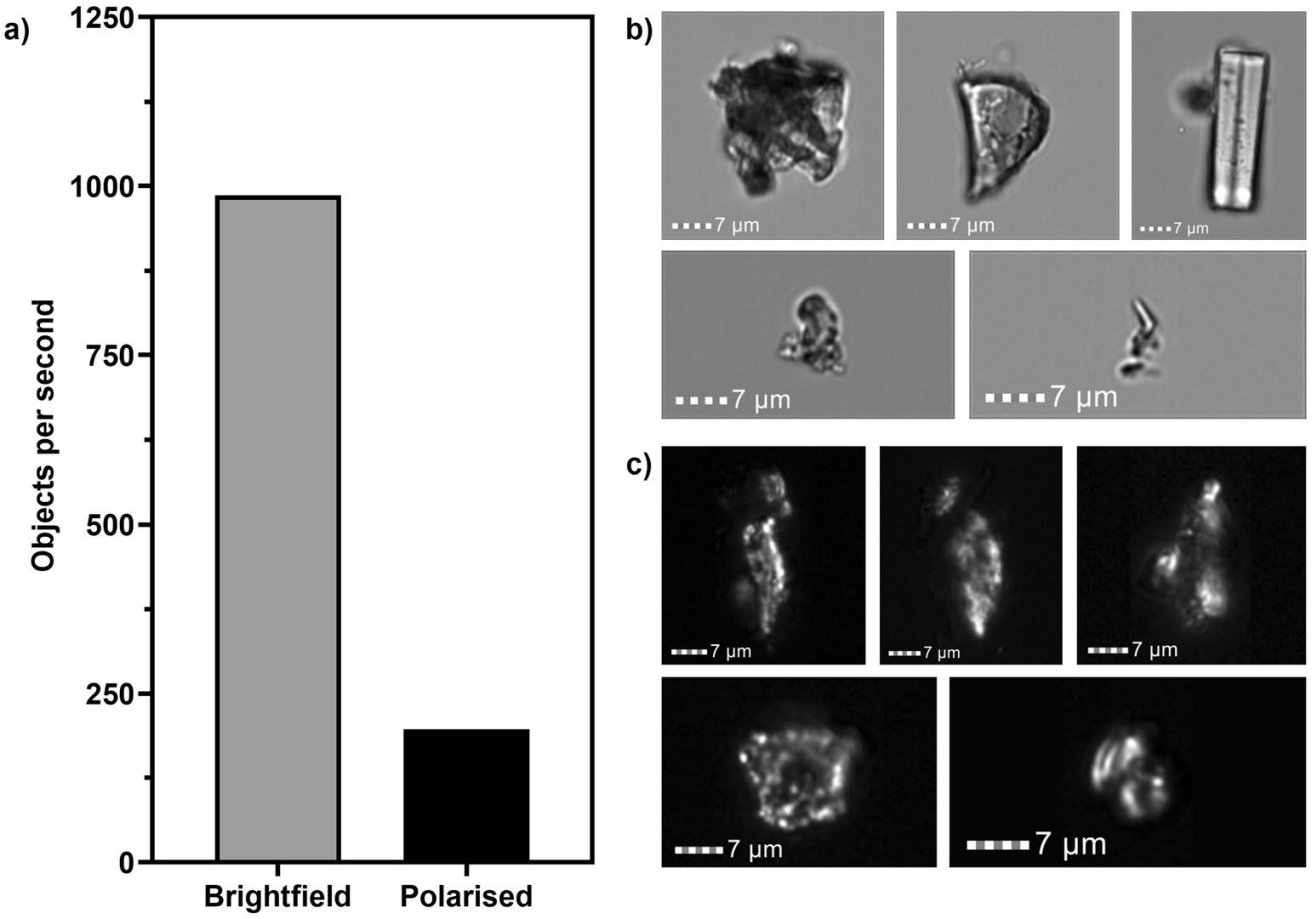
Rate of objects detected without polarisation (brightfield setup) and with polarisation. (**a**) Number of objects detected per second using the ISX without polarisation (brightfield setup) (grey) and with polarisation (black). (**b**) Example images acquired using brightfield setup (channel 4, bandwidth 585/642 nm). (**c**) Example images acquired using polarisation setup (channel 1, bandwidth 457/45 nm).

To rapidly identify potential coccospheres from background marine sediment, untreated marine sediment was analysed under two different configurations: (1) brightfield setup with no polarisation to detect all objects that have a signal above background and (2) with polarisation to exclude all non-birefringent material. Without polarisation, a rate of ~ 1000 objects per second was acquired, compared to ~ 200 objects per second with polarisation (Fig. 2). ISX^+PL^ selectively detected ~ 20% of the total sediment sample as being birefringent, achieving immediate discrimination between birefringent and non-important non-birefringent material.

### Distinguishing coccospheres from birefringent sedimentary background and coccoliths

Birefringent material that may be present in marine sediment includes calcite structures such as coccospheres, coccoliths detached from coccospheres, lorica of tintinnids that incorporate coccoliths into their shell, fragmented foraminifers, and non-biological calcite and non-calcite structure such as quartz. The distinct morphology of coccospheres, compared to other birefringent marine sediment, provided a basis to distinguish them from other birefringent marine sediment using ISX^+PL^.

To distinguish coccospheres from within the identified birefringent population we classified the morphological properties of a positive coccolithophore control and a negative control of marine sediment with CaCO_3_ removed. This morphological data was then used to construct a robust template which could be applied to field samples.

The morphological properties of coccospheres are well documented^36^. Coccospheres are generally less than 30 μm in diameter^36^ and vary in shape from spherical to, in some situations, cylindrical and fusiform^37^. Coccospheres and coccoliths exhibit distinct illumination patterns under cross-polarised light. Coccoliths consist of sub-radial and sub-vertical calcite crystal orientations that appear light and dark in cross-polarised light, with different species exhibiting a specific pattern^38^. A coccosphere that consists of multiple coccoliths and sometimes multiple layers of coccoliths exhibits a greater number and distribution of sub-radial orientated crystals and therefore appears bright under cross-polar illumination, with numerous bright spots.

**Figure 3:**
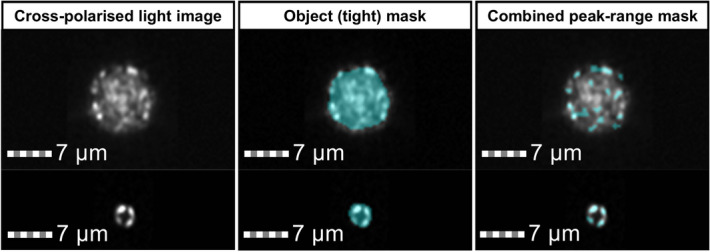
Cross-polarised light images of coccosphere (top) and coccolith (bottom) from cultured coccolithophore sample of the species *G*. *oceanica* with masks overlaid in blue (channel 1, bandwidth 457/45 nm). Object (tight) mask was applied for diameter and circularity value calculations. Combined peak-range mask was applied for bright spot count calculations.

A range of morphological features were considered as criteria to define a coccosphere population. Based on our understanding of the morphological characteristics of coccospheres and their appearance under cross-polarised light, the following features were selected as the criteria most suited for accurate differentiation of coccospheres from non-coccosphere birefringent material:

- Diameter—provides the diameter of the circle that has the same area as the object, based on its mask. The object (tight) mask was used, which uses a set of features to characterise and segment the background and ensures more accurate morphological data (Fig. 3). Diameter is used here as a proxy for size.
- Circularity—measures the degree of the mask’s deviation from a circle. The average distance of the object boundary from its centre is divided by the variation of this distance. A high value describes an object that is close to a circle. This is an ISX-specific feature i.e. not standard circularity. The object (tight) mask was used (Fig. 3). The circularity feature allows the distinct spherical morphology of coccospheres to be quantified.
- Spot Count—identifies and counts regions of high pixel intensity (bright spots). A combined peak-range mask was created; the peak mask identifies all regions of high pixel intensity and the range mask selects the regions of high intensity that are 0.56–555.56 μm^2^ in size (Fig. 3). Spot count is used to characterise surface features and structure. Well-preserved coccoliths will have a spot count of 4 as the R-units produce a pseudo-extinction cross, i.e. 4 points around the radial array of crystals where the birefringence leads to no light passing through the second polarising filter^39^. Coccospheres, which are formed of multiple coccoliths, will therefore have a higher spot count because each coccolith which is approximately perpendicular to the incident light will display 4 bright spots.

**Figure 4:**
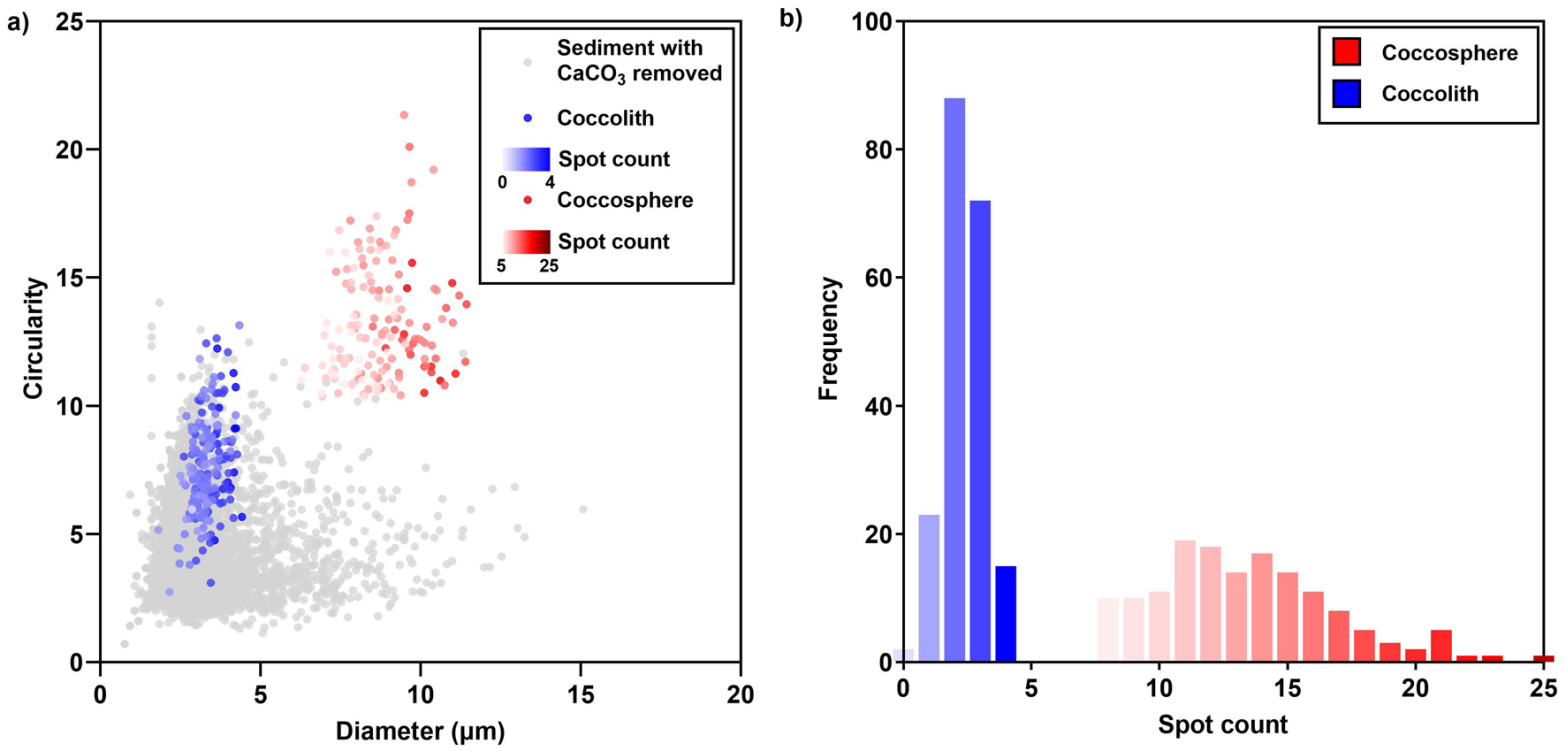
Analysis of selected morphological features of coccospheres, coccoliths and marine sediment with CaCO_3_ removed. (**a**) Diameter and circulatory analysis of 150 coccospheres (red) and 200 coccoliths (blue) from cultured *G*. *oceanica* sample that were visually identified (positive control sample) and 10,000 marine sediment particles from the North Atlantic core 16MC with CaCO_3_ removed (grey) (negative control sample). (**b**) Frequency distribution of spot count values for 150 coccospheres (red) and 200 coccoliths (blue) from cultured *G*. *oceanica* sample (positive control sample).

The positive control (coccospheres of the species *Gephyrocapsa oceanica* obtained from culture) exhibited diameters of 6.273–11.446 μm and high circularity values (> 10.361) (Fig. 4a). These values provided limits for a ‘coccosphere region’. The ‘sediment with CaCO_3_ removed’ sample displayed a highly varied range in diameter (0.752–15.077 μm) and circularity (0.707–14.011), however the majority of these particles had relatively low values for these parameters compared to coccospheres. Only 0.06% of particles from the ‘sediment with CaCO_3_ removed’ plotted in the region where *G*. *oceanica* coccospheres were expressed. This indicated that the morphological properties of coccospheres, specifically diameter and circularity, could be used as a reliable measure of separation from other birefringent marine sediment particles. It is important to note the removal of the calcite component of the marine sediment may include other non-coccolithophore components e.g. non-biological calcite, which may also be expressed in the region of coccospheres.

In a field sample in which multiple coccolithophore species are present, coccospheres and coccoliths may fall within the same shape and size range thus additional criteria are required for separation. Coccospheres were therefore distinguished from coccoliths based on their textural properties using the spot count feature (Fig. 3). The frequency distribution of the number of bright spots in each image showed that coccospheres have 5 or more bright spots and coccoliths have less than 5 bright spots (Fig. 4b). This indicated that coccospheres can be distinguished from coccoliths based on their number of bright spots.

The following decision-making protocol was defined to facilitate visual isolation of coccospheres from marine sediment: firstly, the coccosphere and coccolith population within the birefringent marine sediment was determined based on diameter and circularity, and secondly, coccospheres were distinguished from coccoliths based on bright spot count. Our results indicate this protocol and template can successfully be applied to visually isolate coccospheres from other birefringent and non-birefringent marine sediment (Fig. 4).

### ISX^+PL^ validation

#### Application of ISX^+PL^ to field samples

**Figure 5:**
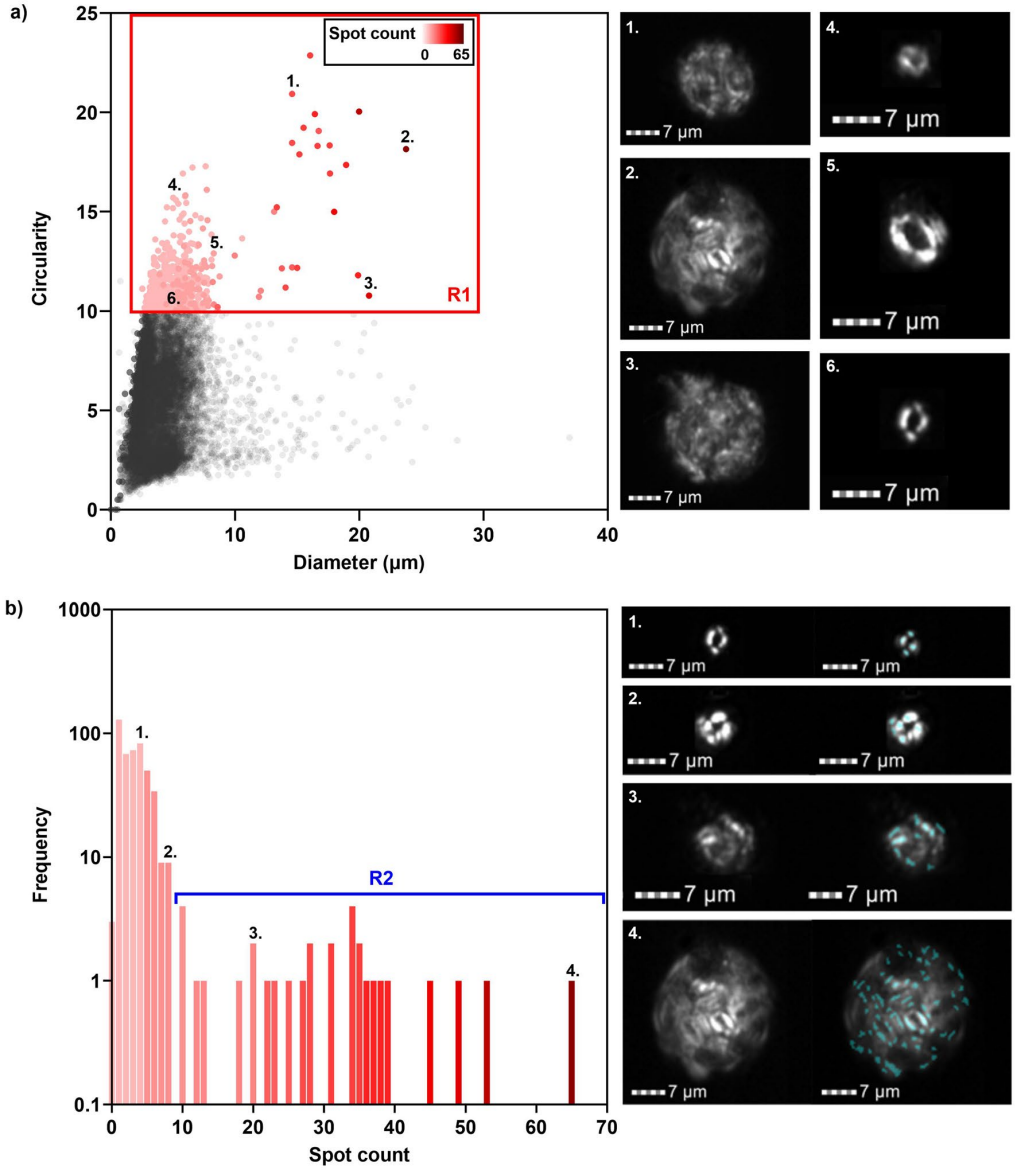
Application of protocol and template to field sample. (**a**) Diameter and circularity analysis of birefringent marine sediment particles from the North Atlantic core 16MC. R1 represents the coccosphere region determined from Fig. 4a and reported species in this geographic region^40^. Representative cross-polarised light images of coccospheres (channel 1, bandwidth 457/45 nm) are presented. (**b**) Spot count frequency distribution analysis of birefringent marine sediment particles from the North Atlantic core 16MC in region R1, Fig. 5a. R2 represents the region where coccospheres are expressed determined via visual analysis of images. Representative cross-polarised light images (channel 1, bandwidth 457/45 nm), and images with combined peak-range mask overlaid, are presented.

To examine the validity of the ISX^+PL^ method we applied the protocol and template (developed using the control samples) to untreated marine sediment from a North Atlantic core. Based upon reported coccosphere geometry measurements for species in this geographical region^36^, we assumed coccospheres to have a diameter in the range 3–30 μm and a high circularity value (≥ 10) (R1, Fig. 5a). Visual analyses of the objects within this region indicated that diameter and circularity provided a good measure of separation of coccospheres from other birefringent material within the sample, however, coccoliths were also expressed in this region. Secondary refinement of this population based on bright spot count was therefore required to define a ‘true’ coccosphere population.

Theoretically, we would expect well-preserved coccoliths to exhibit a spot count value of 4 as the R-units produce a pseudo-extinction cross^39^ (Fig. 3). The number of spots will in part be determined by the orientation of coccoliths as they are imaged and a value less than 4 may also be expected. We observed that a spot count value ≤ 4 allowed discrimination between coccospheres and coccoliths within our positive coccolithophore control sample (Fig. 4b). However, within the field sample, coccoliths exhibited a bright spot count value of up to 10 (Fig. 5b). This may be due to fragmentation of coccoliths or contamination from other birefringent material in the field sample resulting in a greater number of bright spots. A spot count value of ≥ 10 allowed a pure coccospheres population to be defined.

Our positive coccolithophore control sample exhibited coccospheres with a spot count as low as 8 (Fig. 4b), therefore, using a spot count of ≥ 10 to filter out coccoliths may result in a loss of coccospheres from the final determined population. Visual analysis and manual identification of images with a spot count between 5 and 10 would allow all coccospheres within the sample to be distinguished.

**Figure 6:**
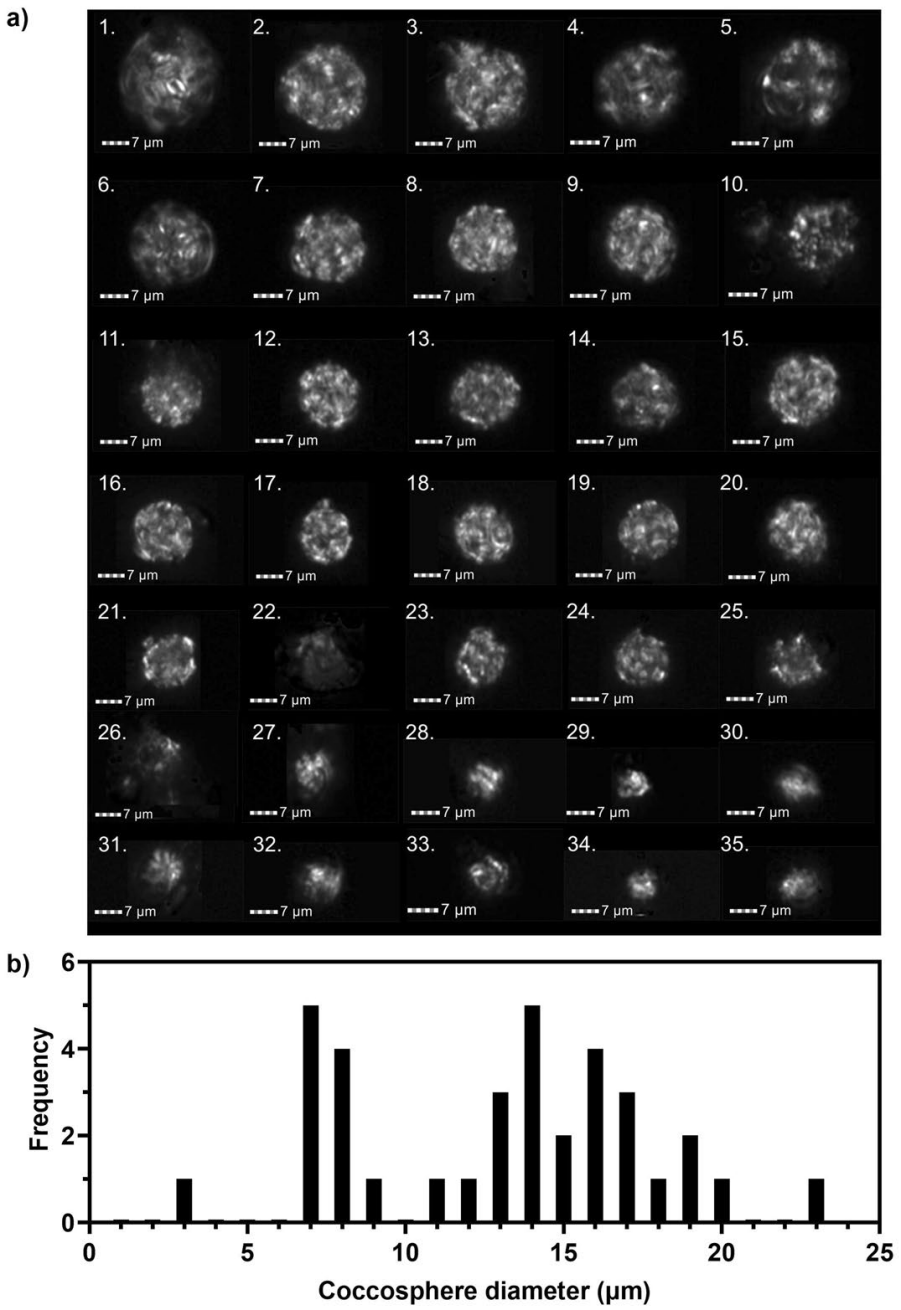
Identified coccosphere population from marine sediment from North Atlantic core 16MC (R2, Fig. 5b). (**a**) Cross-polarised light images (channel 1, bandwidth 470-505 nm) of identified coccosphere population. (**b**) Size distribution of coccosphere diameters.

By combining diameter, circularity (R1, Fig. 5a) and bright spot count (R2, Fig. 5b) a pure population of coccospheres from the North Atlantic core sample was defined (Fig. 6a). A total of 35 coccospheres were identified from 75,000 images of birefringent particles (0.047%), highlighting the sensitivity of the protocol to detect rare events of interest within a bulk sample. Diameters of each coccosphere image were generated from the IDEAS software (Fig. 6b). The size distribution of the defined coccosphere population is consistent with typical sediment samples from this region of the North Atlantic in which the species assemblage is dominated by *Emiliania huxleyi*, *Gephyrocapsa*, *Calcidicus leptoporus* and *Coccolithus pelagicus*^40^.

#### ISX^+PL^ measurement accuracy

ISX^+PL^ measurements were validated by comparison with scanning electron microscopy (SEM) using a cultured coccolithophore sample (Supplementary Fig. S1). There was no significant difference (p = 0.164) between coccosphere diameter measured using ISX^+PL^ (mean = 9.278 μm, standard error of the mean = 0.033 μm) and SEM (mean = 9.488 μm, standard error of the mean = 0.092 μm) as assessed using an unpaired t-test. The narrow discrepancy between our new method and traditional methods (0.21 μm) gives us confidence in the accuracy of coccosphere diameter measurements obtained by ISX^+PL^. The established relationship between coccosphere diameter and cell diameter indicates diameter values obtained via this method can be used as a proxy to reconstruct cell diameter (Fig. 1).

## Discussion

We present a novel technique for the visual isolation, quantification and morphological analysis of coccospheres in sediments, which, unlike traditional microscopy and manual separation^41^, provides high-throughput, rapid and quantitative data acquisition, and removes potential human bias. High-throughput imaging enables the analysis of sediments containing low concentrations of coccospheres in which it would not be feasible to manually ‘hunt’ for coccospheres using traditional microscopy. This technique transforms the scope of micropalaeontological studies of coccolithophores to include geographical regions where nannofossil preservation is rare. Furthermore, application of this method to coccosphere rich sediments enables large datasets and statistically significant results to be generated even for subtly varying parameters. The only sediment not suitable for analysis will be that which is heavily lithophied by the precipitations of mineralogical cement, which cannot be disaggregated. ISX^+PL^ provides rapid data acquisition, greatly increasing the number of positive coccosphere counts that can be achieved in a given amount of time compared to traditional light microscopy. The IDEAS software can generate graphical and statistical information on large data sets and images, removing human measurement bias. Data (images and a range of morphological properties) can be archived and reanalysed to improve reliability.

Applying ISX^+PL^ to investigate the coccosphere fossil record enables cellular level information, such as cell size, to be explored^7^. Existing techniques to reconstruct cell size rely on a positive linear relationship between cell size and coccolith length^42,43^. This provides a reasonable estimate of cell size, however, certain taxa diverge from this trend which can be seen in groups that have large numbers of small coccoliths and the relative cell size is large compared to the coccoliths (e.g. *Kilwalithus*, *Cruciplacolithus primus*, small *Toweius*)^7^. It has also been observed that cell size is a function of growth phase of the population^10^. For example, *Coccolithus* populations display a trend away from the general coccolith length to cell size relationship at different phases of cell division; cells prior to division are large, recently divided cells are smaller, and undividing cells in the stationary phase are the largest size^7,10^. Using ISX^+PL^, we can begin to move closer to a direct measurement of coccolithophore community cell size by exploiting the coccosphere fossil record to reconstruct cell size from coccosphere measurements (Fig. 1).

ISX^+PL^ enables rapid processing of down-core sediment samples or high spatial coverage from surface sediments allowing detection of high-resolution temporal or spatial changes in coccosphere size, and estimates of cell size. Cell size is an important parameter that enables us to examine the evolution and ecology of coccolithophore communities^11,44,45^. Temporal trends in cell size provide a record of micro- and macroevolution, for example, the reduction in size (stunting or dwarfing) immediately following extinction (Liliput effect^46^) and the general radiation towards a larger cell size following speciation events (Cope’s Rule^47^). Cell size is a fundamental master trait that is intricately linked with physiological and ecological processes^9^. Coccosphere and cell geometry are directly linked with growth phase in the fossil record with cells undergoing rapid cell division (growth phase) typically smaller than cells dividing slowly (stationary phase)^10^. As growth phases describe states of rapid or slowed growth, with due considerations, cell size may provide an insight into the ecological “fitness” and subsequent evolution of coccolithophore populations. Cell size can therefore be used as a record of the response of species and communities to palaeoceanographic and palaeoclimatic variability. Our current understanding of coccolithophore response to palaeoceanographic and palaeoclimatic variability is largely based on short term laboratory culture experiments which have yielded complex and often contradictory responses between and even within species^48,49^. Laboratory studies lack the complex interplay and interactive effect of multiple stressors^50–52^, neglect ecological processes such as competition^53^ and short-term experiments omit potential adaptive evolution^54,55^. The application of ISX^+PL^ to reconstruct coccolithophore response and adaptive capacity to climate change over geological time has the potential to overcome the constraints of laboratory investigations and reveal critical information regarding how these complex systems may respond to future change.

An additional potential application of this method is the use of IXS^+PL^ derived cell size estimates for calculations of palaeoenvironmental proxies that analyse the geochemistry of coccolithophore organic matter or coccolith calcite. Such calculations are dependent on cell size due to the influence of cell size and growth rates on the partitioning of elements and isotope involved in organic and inorganic carbon production^56^. Cell size and growth rates are critical for reconstructions of palaeo-CO_2_ concentrations and palaeotemperature from stable carbon isotope analysis within sediment-preserved coccolithophore alkenones^42^, Sr/Ca and Mg/Ca ratios in coccolith calcite^57–60^ and stable isotopes of carbon and oxygen obtained from coccolith calcite^56,61–63^.

Despite coccospheres representing a rare opportunity to reconstruct cell size from the past, the resultant data may be difficult to interpret due to complexities associated with preservation^4^. Coccospheres are particularly vulnerable to alteration and/or disintegration which can occur at any point from cell death to analysis of the sample, through both physical and chemical processes^7^. Coccosphere remains are therefore not necessarily a direct image of the former living community, but one that is altered in species composition and reduced in abundance^64^. Among the ∼ 280 types of coccosphere known from modern plankton, only 102 are known to exist in Holocene sediments, with 45 of these being very rare^4^. Therefore, more than 60% of the diversity is erased and leaves no fossil record. The vast majority of coccospheres we observe in the fossil record are structurally strong species that produce interlocking placolith-type coccoliths^7^. The record of species with murolith-type coccoliths, that are more susceptible to disintegration, is absent. Size related preservation biases also exist with very small or very large coccospheres vulnerable to disarticulation^4^. Coccospheres that do reach the seafloor may undergo secondary dissolution or overgrowth giving a false coccosphere and cell size signal. Furthermore, preservation potential may not be consistent throughout time at the same site due to changing environmental conditions that enhance or diminish preservation potential^4^, and hence change coccosphere size.

Preservation-related factors should be carefully considered in the interpretation of coccosphere data. Clay-rich hemipelagic but organic-carbon-poor sediments that favour preservation will limit preservation-bias and provide a record that best represents the former living coccolithophore community^7^. With a good understanding of site-specific preservation bias, interpretations of the fossil record can be made to comprehend the relationships between the living and fossilised coccolithophore realms.

The ISX is a powerful tool that compares well with visual microscopy analysis^65^. The object (tight) mask used for diameter and circularity calculations and high pixel resolution (0.33 μm^2^) ensures that coccosphere measurements are accurate. Our validation of ISX^+PL^ derived coccosphere diameter values with SEM measurements demonstrates that this technique achieves accuracy that is comparable with traditional SEM methods (Supplementary Fig. S1). We are confident biases are not introduced from ISX^+PL^ measurements, however, the following considerations should be made when using data generated using ISX^+PL^.

The coccosphere size v. cell size relationship we present (Fig. 1) holds true for the dominant well-fossilised heterococcolith families, the *Coccolithaceae*, *Calcidiscaceae* and *Noelaerhabdaceae*, however, in certain taxa there will be divergence from this trend due to taxon-specific variation in the number of coccoliths per coccosphere, coccolith shape and packing and arrangement of coccoliths^7,10^. Within the species examined in this study, there is significant difference between species-specific coccosphere-cell trends and therefore this variation presents possible errors in reconstructing cell size from coccosphere size. It is therefore important to consider the taxonomic make-up of the sample and define a relationship representative of the species assemblage to ensure cell diameter estimates are accurate.

Possible error may arise when defining the coccosphere population from marine sediment. The main families considered in this study exhibit bright, spherical coccospheres under polarised light, however, it is important to note this is not always the case for fossil taxon. To apply this method to marine sediments of different taxonomic make-up from different time periods or geographical regions, characterising morphologically variable species will aid the development of robust and reliable templates for coccosphere identification. Feature value limits are critical in defining coccosphere populations. The template used in this study presents criteria for achieving a pure coccosphere population, however we highlight possible exclusion of coccospheres from this population. To separate coccoliths a spot count value ≥ 10 was applied to our field sample (Fig. 5b), yet coccospheres in the control sample exhibited a spot count as low as 8 (Fig. 4b), therefore, coccospheres may have been omitted from the pure coccosphere population. Furthermore, in the application of this template to marine sediments of different lithologies and compositions, any birefringent material that is morphologically similar to coccospheres may skew signals and introduce error in the defined coccosphere population. Imaging flow cytometry offers an advantage over traditional flow cytometry in that each object is imaged and using the IDEAS software, samples can be classified and concentrated into smaller populations of interest. Utilising these features, errors arising when defining a coccosphere population can be quantified and overcome by manual visual identification of a concentrated sample of images, enabling all coccospheres to be defined or sediment contamination to be excluded.

ISX^+PL^ achieves a visual sort but given rapid advances in imaging flow cytometry, the scope for physically sorting coccospheres from sediment, which would enable other analyses such as electron microscopy for higher resolution taxonomic classification and basic elemental analysis, should not be discounted. Physical sorting of coccospheres opens up an array of options for downstream geochemical analysis^26^, which at present can only be undertaken on individual coccoliths^41,66^ or bulk sediment.

The weight of individual coccoliths can be estimated from their birefringence^31^. Building on from the approach of Beaufort et al.^67^, von Dassow et al.^68^ demonstrate the potential for polarisation-sensitive flow cytometry to provide a method for rapidly assessing the calcification state of individual phytoplankton cells by correlating the ratio of orthogonally polarised forward scattered light to parallel polarised forward scattered light with coccosphere calcite mass. Balch et al.^69^ used flow cytometry with linear polarizers to sort ^14^C‐labelled *E*. *huxleyi* cells and estimate the rate of synthesis of coccoliths and number of layers produced. The current optical configuration of the ISX is unable to measure parallel polarised forward scattered light, however, development of new calibration could be an improvement on traditional flow cytometry techniques as complicating factors such as coccosphere size could be measured and corrected for.

We have presented a novel technique (ISX^+PL^) to rapidly and reliably visually isolate and analyse the morphology of coccospheres from marine sediment. By combining, for the first time, imaging flow cytometry and cross-polarised light, exploiting the optical and morphological characteristics of coccospheres has allowed the identification of these rare nannofossils, preserved within marine sediments. This approach transforms the speed and accuracy with which coccospheres can be detected and overcomes limitations associated with traditional microscopy, providing rapid data acquisition and analysis and removes potential human bias. The results we present here demonstrate a step towards unlocking otherwise inaccessible information afforded by fossil coccospheres.

## Methods

### Sample preparation for ISX^+PL^

Three samples were analysed for the development of the ISX^+PL^ method, a field sample, a positive and a negative control. The field sample consisted of marine sediment from the North Atlantic core EN539-16MC (61° 29 N, 19° 32 W, 2311 m water depth). A sample was taken at 12–12.5 cm depth (estimated age is 1939 based on combined ^210^Pb, radiocarbon and water content^70^) and wet sieved at 63 μm using deionised Milli-Q water and the fine-fraction was retained. ~ 100 mg of sediment was agitated with 15 ml deionised Milli-Q water then filtered through a 100 μm cell strainer to remove aggregates. A 100 μl subsample was transferred into a centrifuge tube.

For the positive control sample, we used batch cultured *Gephyrocapsa oceanica* (RCC 1314), obtained from the Roscoff Culture Collection. Additional information about the strain RCC 1314 can be found at http://roscoff-culture-collection.org/. A 100 μl subsample was transferred into a centrifuge and analysed without any further sample preparation. In the IDEAS software, 150 coccosphere images and 200 coccolith images from the positive control were visually identified and used for analysis. For the negative control sample of marine sediment from the North Atlantic core 16MC with CaCO_3_ removed by acid addition^71^, the sediment was initially processed using the same method as the field sample: it was sieved at 63 μm and the fine-fraction was retained. However, the sediment was then subjected to 10% HCl to dissolve CaCO_3_ (and hence any coccospheres), which was visually confirmed via light microscopy. Sediments were then washed to neutralise samples (through centrifugation technique) and the sediment was suspended in deionised Milli-Q water before filtering through a 100 μm cell strainer to remove aggregates. A 100 μl subsample was transferred into a new centrifuge tube.

Prior to analysing the samples on the ISX, all samples were vortexed to re-suspend particles.

### Data acquisition using ISX^+PL^

Data was acquired using ISX (ImageStream Mk II; Luminex Corp. Seattle, US), configured with a six-channel system, 405 nm, 488 nm, 642 nm and 758 nm lasers, and brightfield light. The ISX was uniquely fitted with two bespoke light polarising filters (WP12L-VIS, 420–700 nm) situated in the collection path of the brightfield illumination system. The first filter polarises the light such that the wave vibrates along a single plane in a vertical direction. The second polarising filter is rotated at 90° to block all illumination apart from light moving in a horizontal direction. Birefringent material, such as calcite, splits the incident polarised light into two orthogonally polarised rays, thus allowing light to pass through the second polariser and particles to be visualised. The ISX^+PL^ therefore selectively detects birefringent structures only.

The ISX was configured to 60X magnification (0.33 μm^2^ pixel resolution). The objective lens used was Olympus UPLFN60X 0.90NA. Low flow speed was used for high sensitivity. 1 μm polystyrene speed beads were used for calibration of the flow and continuous focus of the ISX. During acquisition, all lasers were turned off and interrogated by the brightfield light. The polarising filter arrangement greatly reduces transmitted light; therefore, all brightfield LEDs were set at full intensity to illuminate particles in the flow stream. Cross-polarised light emitted by samples at different spectral bands were detected on a six-channel sensor. Images from channel 1 (457/45 nm bandwidth), were used for analysis.

For brightfield analysis in “Coccosphere detection using birefringent properties”, the polarising filters were removed from the collection path of the brightfield illumination system and the sample was interrogated by brightfield light only. Speed beads were discriminated based on their distinct diameter and aspect ratio and eliminated from both brightfield and polarised data files before calculating objects detected per second.

### Data analysis using ISX^+PL^

Data analysis was performed using IDEAS software (Luminex Corp) which allows analysis of a range of morphological features, fluorescence, and scatter of light. All morphological data is derived from ‘masks’, which are regions of the image defined as meeting certain criteria and are used for displaying feature-value calculations. High-quality, focused images were identified using Gradient RMS (root mean square), which measures the sharpness of an image. Objects with a Gradient RMS value greater than 15 were selected for further analysis and unfocused objects excluded. Aspect ratio was used to distinguish single objects from images containing multiple objects. Single objects have a relatively higher aspect ratio and therefore objects with an aspect ratio greater than 0.75 were selected for further analysis.

### SEM validation

The diameters of coccospheres from batch cultured *Gephyrocapsa oceanica* (RCC 1314) were measured using ISX^+PL^ and SEM. Both techniques were performed within 24 h.

For ISX analysis, a 100 μl subsample was transferred into a centrifuge tube. The ISX was configured with the polarising arrangement as described in “Data acquisition using ISX^+PL^” and images of all objects in the sample were acquired. In the IDEAS software, 1000 images were visually identified as coccospheres and using the tight (object) mask, mean coccosphere diameter was computed.

For SEM analysis, coccosphere samples were passed through a 0.2 μm polycarbonate filter using a mild vacuum and the filter immediately transferred into deionized water. After a 2-min rinse, the filter was air dried then mounted onto an aluminium sample pin fitted with a sticky carbon tab. The samples were coated with a 10 nm layer of gold/palladium (80/20) using a sputter coater (Q150T ES, Quorum Technologies Ltd, Laughton, UK). Samples were imaged using a Zeiss GeminiSEM 500 (Carl Zeiss Ltd, Cambridge, UK) operated at 5 kV. For quantifying the mean calliper diameter of coccospheres the polycarbonate filter was scanned systematic uniform random and the calliper diameter of 50 intact coccospheres measured during the live scan along the horizontal and vertical orientation related to the field of view.

### Statistical analysis

Statistical analyses were performed in GraphPad Prism (version 8; GraphPad Software, Inc., USA). Simple linear regression analysis was used to estimate parameters to construct a linear regression equation to predict cell size from coccosphere size. A method equivalent to an Analysis of Covariance (ANCOVA) was conducted to determine if linear regression slopes for different species were significantly different from each other. An unpaired t-test was used for statistical comparison of the coccosphere size populations measured using ISX^+PL^ and SEM.

## Supplementary information

Supplementary Information.

## Data Availability

The datasets generated (raw images) during the current study are available in the Zenodo repository; https://doi.org/10.5281/zenodo.3979045.
