# Supplementary material for: A new method for isolating and analysing coccospheres within sediment
Source: Sci Rep. 2020 Nov 26;10:20727. doi: 10.1038/s41598-020-77473-5 (PMC7692543; doi:10.1038/s41598-020-77473-5)

Supplementary Information

Title: A New Method for Isolating and Analysing Coccospheres within Sediment

Authors: Beth Langley, Paul Halloran, Ann Power, Rosalind Rickaby, Prabhjoat Chana, Poppy Diver, David Thornalley, Christian Hacker and John Love

Figure S1: Frequency plot of coccosphere size populations measured using ISX^{+PL} (grey, n = 1000) and SEM (red, n = 50).

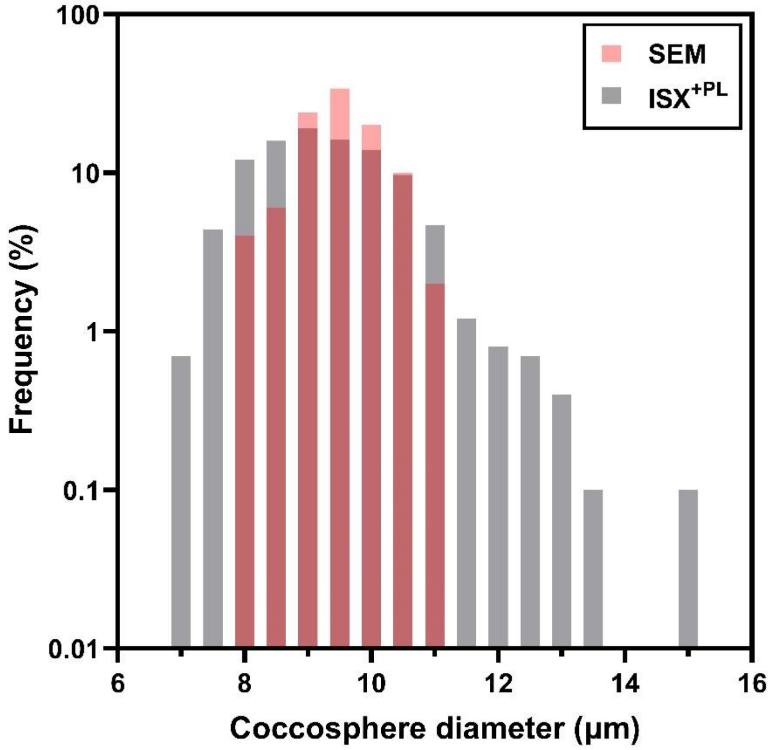

Supplement: Supplementary file 1 — Supplementary Information. [file 41598_2020_77473_MOESM1_ESM.pdf]
